# Supplementary material for: Mapping the coevolution, leadership and financing of research on viral vectors, RNAi, CRISPR/Cas9 and other genomic editing technologies
Source: PLoS One. 2020 Apr 15;15(4):e0227593. doi: 10.1371/journal.pone.0227593 (PMC7159216; doi:10.1371/journal.pone.0227593)
Supplement: S1 Table — (PDF) [file pone.0227593.s001.pdf]

Clusters	Number of papers	Number of inter-citations	Percentage of papers of each one of the studied technologies	Leading research institutions (Institutions with more papers in the top ten percent of papers with the highest indegree within each cluster)	Leading funding agencies (Agencies with more papers in the top ten percent of papers with the highest indegree within each cluster)	Stage of the investigation<br><br>Type of leading research organizations and funding entities
1966-2000						
C1-2000: "Retroviral vectors for gene expression; hematopoietic cells"	438	1352	Viral vectors: 98.5%<br><br>meganucleases: 1.4%	National Institutes of Health USA<br><br>University of Southern California<br><br>Children's Hospital Los Angeles	National Institutes of Health USA	Invention<br><br>Academic institutions with public funding
C2-2000: "Recombinant adeno-associated virus vectors"	296	2241	viral vectors: 100%	University of North Carolina at Chapel Hill<br><br>University of Pennsylvania	National Institutes of Health USA	Invention<br><br>Academic institutions with public funding
C3-2000: "Immune response to adenoviral vectors"	248	638	viral vectors: 100%	Baylor College of Medicine<br><br>Howard Hughes Medical Institute	National Institutes of Health USA	Invention<br><br>Academic institutions with public funding
C4-2000: "Molecular biology of adeno-associated virus; Rep78"	229	2030	viral vectors: 100%	University of Florida	National Institutes of Health USA	Basic research<br><br>Academic institutions with public funding
C5-2000: "lentiviral vectors; lentiviral vectors pseudotyped with vesicular stomatitis virus G glycoprotein"	146	480	viral vectors: 100%	Salk Institute for Biological Studies<br><br>University of California San Diego	National Institutes of Health USA	Improvement<br><br>Academic institutions with public funding
C6-2000: "Targeted retroviral vectors"	128	270	viral vectors: 100%	No leading research institution identified	National Institutes of Health USA	Improvement<br><br>Leading research institutions and funding agencies not identified
C7-2000: "Mechanisms and function of post-transcriptional gene silencing; Plants; C. elegans"	48	142	RNAi: 100%	No leading research institution identified	No leading founding agency identified	Basic research<br><br>Leading research institutions and funding agencies not identified
C8-2000: "Homing endonucleases: structure and function"	45	225	meganucleases: 100%	Fred Hutchinson Cancer Research Center<br><br>University of Washington	No leading founding agency identified	Basic research<br><br>Academic institutions
2001-2005						
C1-2005: "AAV vectors; AAV serotypes"	419	1530	Viral vectors: 99.8%<br><br>RNAi/viral vectors combined: 0.2%	University of Florida<br><br>National Institutes of Health USA<br><br>University of Pennsylvania	National Institutes of Health USA	Application<br><br>Academic institutions with public funding
C2-2005: "RNA interference (RNAi) pathway; Drosophila"	280	1884	RNAi: 99.2%<br><br>Viral vectors: 08%	Cold Spring Harbor Laboratory<br><br>University of Massachusetts Medical School<br><br>Howard Hughes Medical Institute	National Institutes of Health USA	Basic research<br><br>Academic institutions with public funding
C3-2005: "Efficient transduction of mammalian cells by lentiviral vectors"	313	769	Viral vectors: 99.4%<br><br>RNAi: 0.4%	University of Turin<br><br>Fondazione del Piemonte per l'Oncologia – IRCCS<br><br>Salk Institute for Biological Studies<br><br>University of Geneva	National Institutes of Health USA	Improvement<br><br>Academic institutions with public funding
C4-2005: "RNA interference in mammalian cells"	252	947	RNAi: 90.1%<br><br>RNAi/viral vectors combined: 3.2%<br><br>Viral vectors: 6.7%	Cold Spring Harbor Laboratory	National Institutes of Health USA	Invention<br><br>Academic institutions with public funding
C5-2005: "Genome-wide analysis of gene function by high-throughput RNAi screening"	155	393	RNAi: 97.4%<br><br>RNAi/viral vectors combined: 1.3%<br><br>Viral vectors: 1.3%	University of Cambridge<br><br>Cold Spring Harbor Laboratory<br><br>Howard Hughes Medical Institute<br><br>Harvard University	Wellcome Trust	Research tool<br><br>Participation of philanthropic foundations or for-profit organizations
C6-2005: "Helper-dependent adenoviral vectors; toxicity of first-generation adenoviral vectors"	144	338	Viral vectors: 100%	Baylor College of Medicine<br><br>University of Pennsylvania<br><br>The Wistar Institute	National Institutes of Health USA	Application<br><br>Academic institutions with public funding
C7-2005: "Site-specific DNA activity of the AAV Rep protein"	108	238	Viral vectors: 100%	Icahn School of Medicine at Mount Sinai	National Institutes of Health USA	Basic research<br><br>Academic institutions with public funding
C10-2005: "DNA binding site/specificity of homing endonucleases"	59	183	Meganucleases: 84.7%<br><br>ZFNs: 15.3%	Fred Hutchinson Cancer Research Center<br><br>Université Laval<br><br>University of Washington Seattle	National Institutes of Health USA	Basic research<br><br>Academic institutions with public funding
2006-2010						
C1-2010: "AAV vectors; AAV serotypes; Cell/tissue tropism"	361	890	Viral vectors: 96.4%<br><br>RNAi/viral vectors combined: 1.4%<br><br>RNAi: 2.2%	University of Pennsylvania<br><br>University of Florida<br><br>University of North Carolina Chapel Hill	National Institutes of Health USA	Application<br><br>Academic institutions with public funding
C2-2010: "Induction of RNAi by double-stranded RNA (dsRNA)"	231	507	RNAi: 99.2<br><br>Viral vectors: 0.8%	CSIC Instituto de Biologia Molecular de Barcelona<br><br>University of California System	National Institutes of Health USA<br><br>Wellcome Trust	Basic research<br><br>Participation of philanthropic foundations or for-profit organizations
C3-2010: "Inhibition of virus replication; Small interfering RNAs (siRNAs)"	218	379	RNAi: 88.5%<br><br>RNAi/viral vectors combined: 6%<br><br>Viral vectors: 5.5%	Alnylam Pharmaceuticals Inc	National Institutes of Health USA	Application<br><br>Participation of philanthropic foundations or for-profit organizations
C4-2010: "short hairpin RNAs (ShRNA)"	207	310	RNAi: 82.2%<br><br>Viral vectors: 10.1%<br><br>RNAi/viral vectors combined: 7.7%	University of California System	National Institutes of Health USA	Improvement<br><br>Academic institutions with public funding

C5-2010: "Genome-wide RNAi screens (Mostly in Drosophila)"	192	387	RNAi: 98%<br><br>RNAi/viral vectors combined: 1%<br><br>Viral vectors: 1%	Harvard University<br><br>Howard Hughes Medical Institute<br><br>VA Boston Healthcare System	National Institutes of Health USA<br><br>Howard Hughes Medical Institute	Research tool<br><br>Academic institutions with public funding
C6-2010: "Self-inactivating retroviral vectors; Insertional mutagenesis"	181	433	Viral vectors 97.8%<br><br>RNAi: 2.2%	Cincinnati Children's Hospital Medical Center<br><br>Hannover Medical School<br><br>University of Cincinnati<br><br>Helmholtz Association	National Institutes of Health USA	Improvement/Application<br><br>Academic institutions with public funding
C7-2010: "Lentiviral vectors for immunization"	133	322	Viral vectors: 97.5%<br><br>RNAi: 2.5%	University College London	National Institutes of Health USA	Application<br><br>Academic institutions with public funding
C8-2010: "Adenoviral vectors; adeno-associated; pre-clinical gene therapy"	104	148	Viral vectors: 96.1%<br><br>RNAi: 2.9<br><br>RNAi/viral vectors combined: 1%	Baylor College of Medicine<br><br>Cedars-Sinai Medical Center<br><br>David Geffen School of Medicine at UCLA	National Institutes of Health USA	Application<br><br>Academic institutions with public funding
C9-2010: "Genome editing driven by zinc-finger nucleases (ZFNs)"	85	502	ZFNs: 78.7%<br>Meganucleases: 7.1%<br>Viral vectors: 11.8%<br>RNAi: 1.2%<br>ZFNs/viral vectors combined: 1.2%	Sangamo BioSciences Inc	National Institutes of Health USA	Invention<br><br>Participation of philanthropic foundations or for-profit organizations
C12-2010: "Engineered homing endonucleases; I-DmoI as scaffold for protein engineering; meganucleases"	64	160	Meganucleases: 90.6 %<br><br>Viral vectors 9.4%	Fred Hutchinson Cancer Research Center<br><br>University of Washington Seattle	National Institutes of Health USA	Invention<br><br>Academic institutions with public funding
C14-2010: "CRISPR as a component of the antiviral system of prokaryotes"	53	256	CRISPR: 100%	Danisco France SAS<br>Université Laval	National Institutes of Health USA	Basic research<br><br>Participation of philanthropic foundations or for-profit organizations
2011-2015						
C1-2015: "RNA-guided genome editing via Cas9"	534	4809	CRISPR: 89%<br><br>TALENs: 2.6%<br><br>RNAi: 2.6%<br><br>Genome editing platforms combined: 2.2%<br><br>Viral vectors 1.1%<br><br>ZFNs: 0.9%<br><br>Meganucleases: 0.9%<br><br>Genome editing/viral vectors combined: 0.7%	Broad Institute<br><br>Harvard University<br><br>Massachusetts Institute of Technology<br><br>Howard Hughes Medical Institute	National Institutes of Health USA<br>Damon Runyon Foundation<br><br>Howard Hughes Medical Institute<br><br>Klingestein Foundation<br><br>Searle Scholars Foundation<br><br>Simons Foundation<br><br>W M Keck Foundation	Invention<br><br>Participation of philanthropic foundations or for-profit organizations
C2-2015: "RNAi for Insect Control; delivery of dsRNA for pest control"	413	800	RNAi: 98.3%<br><br>RNAi/viral vectors combined: 0.5%<br><br>Viral vectors: 1.2%	University of California System<br><br>University of Edinburgh<br><br>Harvard University<br><br>National Centre of Scientific Research Demokritos	National Institutes of Health USA<br><br>Research Foundation - Flanders (FWO)<br><br>National Natural Science Foundation of China<br><br>Wellcome Trust	Application<br><br>Participation of philanthropic foundations or for-profit organizations
C3-2015: "Genome editing using TALENs and ZFNs"	404	1525	TALENs: 41.1%<br><br>ZFNs: 34.4%<br><br>Viral vectors: 8.2%<br><br>CRISPR: 5.4%<br><br>Meganucleases: 4.2<br><br>Genome editing platforms combined: 3.2%<br><br>RNAi: 2.7%<br><br>Genome editing/viral vectors combined: 0.7%	Harvard University<br><br>VA Boston Healthcare system<br><br>Massachusetts General Hospital	National Institutes of Health USA<br><br>National Science Foundation USA	Invention (TALENs)/ improvement (ZFNs)<br><br>Academic institutions with public funding
C4-2015: "AAV vectors; AAV serotypes; Clinical applications/trials"	361	756	Viral vectors: 95.8<br><br>RNAi: 2.5%<br><br>RNAi/viral vectors combined: 1.7%	State University System of Florida<br><br>University of North Carolina<br><br>University of California System<br><br>University of Pennsylvania	National Institutes of Health USA	Improvement<br><br>Academic institutions with public funding
C5-2015: "CRISPR RNA (crRNA); CRISPR/Cas mediated adaptive immunity system; crRNA-guided Cascade complex"	329	2909	CRISPR: 100%	United States Department of Energy<br><br>Wageningen University Research<br><br>Howard Hughes Medical Institute<br><br>Lawrence Berkeley National Laboratory	National Institutes of Health USA<br><br>National Science Foundation USA	Basic research<br><br>Academic institutions with public funding
C6-2015: "Lentiviral vector; clinical trials"	204	329	Viral vectors: 97.5%<br><br>RNAi: 1.5%<br><br>RNAi/viral vectors combined: 1%	Fundazione Telethon<br><br>Vita Salute San Raffaele University<br><br>Helmholtz Association, German Cancer Research Center<br><br>Institut national de la santé et de la recherche médicale<br><br>Ruprecht-Karls-Universität Heidelberg, University of London	Fundazione Telethon<br><br>European Union<br><br>German Research Foundation	Innovation<br><br>Academic institutions with public funding

C7-2015: "RNAi Screening"	183	255	RNAi: 95.7%<br><br>Viral vectors: 1.1%<br><br>CRISPR: 0.5%<br><br>TALENs: 0.5%<br><br>RNAi/viral vectors combined: 2.2%	Harvard University<br><br>Helmholtz Association<br><br>German Cancer Research Center<br><br>Howard Hughes Medical Institute<br><br>Memorial Sloan Kettering Cancer Center	National Institutes of Health USA<br><br>European Union	Research tool<br><br>Academic institutions with public funding
C9-2015: "Genome Engineering of Drosophila and Caenorhabditis with the CRISPR/Cas9"	83	429	CRISPR: 68.7%<br><br>RNAi: 20.5%<br><br>TALENs: 8.4%<br><br>RNAi/viral vectors combined: 1.2%<br><br>ZFNs: 1.2%	Harvard University	National Institutes of Health USA	Improvement<br><br>Academic institutions with public funding
C10-2015: "Engineered LAGLIDADG homing endonucleases (LHEs)"	53	113	Meganucleases: 88.7<br><br>Viral vectors: 7.5%<br><br>ZFNs: 3.8%	University of Washington Seattle<br><br>Fred Hutchinson Cancer Center	National Institutes of Health USA	Improvement<br><br>Academic institutions with public funding
C11-2015: " CRISPR/Cas and TALENs systems as tools for genome engineering in plants"	42	172	CRISPR: 76.2<br><br>TALENs: 11.9%<br><br>ZFNs: 9.5%<br><br>RNA1: 2.4	Chinese Academy of Science	Chinese Academy of Science<br><br>National Natural Science Foundation of China	Application<br><br>Academic institutions with public funding
2016-2019						
C1-2019: "Cas9-mediated correction of diseases; delivery of CRISPR/Cas9"	349	804	CRISPR: 78.2%<br><br>Viral vectors: 6.9%<br><br>Genome editing/viral vectors combined: 6%<br><br>RNAi: 3.7%<br><br>TALEN: 1.4%<br><br>ZFNs: 1.4%<br><br>Genome editing platforms combined: 1.2%<br><br>Meganucleases 1.1%	University of California System<br><br>Harvard University<br><br>Chinese Academy of Science	National Institutes of Health USA<br><br>National Natural Science Foundation of China<br><br>National High Technology Research Program of China	Application<br><br>Academic institutions with public funding
C2-2019: "Crispr-cas9-based genetic screens"	294	729	CRISPR: 92.8%<br><br>RNAi: 3.7%<br><br>CRISPR/RNAi combined: 1.4%<br><br>TALENs: 1.4%<br><br>Viral vectors: 0.7%	Harvard University<br><br>Broad Institute<br><br>Massachusetts Institute of Technology	National Institutes of Health USA<br><br>National Science Foundation USA	Research tool<br><br>Academic institutions with public funding
C3-2019: "Cas9 off-target activity; Cas9 cleavage"	284	764	CRISPR: 95.6%<br><br>TALEN: 1.8%<br><br>RNAi: 0.7%<br><br>Viral vectors: 0.7%<br><br>ZFNs: 0.4%<br><br>Genome editing/viral vectors combined: 0.4%<br><br>Genome editing platforms combined: 0.4%	University of California System<br><br>Harvard University<br><br>Howard Hughes Medical Institute	National Institutes of Health USA<br><br>National Science Foundation USA	Improvement/application<br><br>Academic institutions with public funding
C5-2019: "Mechanisms of crispr-cas imunity; Crispr-cas immunity systems"	204	772	CRISPR: 100%	University of California Berkeley<br><br>Rockefeller University<br><br>Howard Hughes Medical Institute	National Institutes of Health USA<br><br>National Science Foundation USA	Basic research<br><br>Academic institutions with public funding
C6-2019: "CRISPR/Cas systems as tools for genome engineering in plants"	186	388	CRISPR: 91%<br><br>TALENs: 3.2%<br><br>Genome editing platforms combined: 1.6%<br><br>RNAi: 1.6%<br><br>ZFNs: 1.6%<br><br>CRISPR/RNAi combined: 0.5%<br><br>Viral vectors: 0.5%	Chinese Academy of Science<br><br>Purdue University<br><br>University of Queensland	National Natural Science Foundation of China<br><br>Chinese Academy of Science	Improvement/application<br><br>Academic institutions with public funding
C7-2019: "Adeno-associated virus (AAV) vectors"	178	211	Viral vectors: 87.1%<br><br>CRISPR: 11.2%<br><br>RNAi: 1.1%<br><br>CRISPR/RNAi combined: 0.6%	University of Florida<br><br>University of North Carolina Chapel Hill	National Institutes of Health USA<br><br>National Natural Science Foundation of China	Improvement/application<br><br>Academic institutions with public funding
C8-2019: "CRISPR-associated endonuclease in Prevotella and Francisella 1 (cpf1)"	175	574	CRISPR: 95.5%<br><br>RNAi: 1.7%<br><br>Genome editing platforms combined: 1.1%<br><br>Viral vectors: 1.1%<br><br>RNAi/viral vectors combined: 0.6%	Harvard University<br><br>Broad Institute<br><br>Massachusetts Institute of Technology	National Institutes of Health USA<br><br>Vallee Foundation<br><br>New York Stem Cell Foundation<br><br>Simons Foundation	Improvement<br><br>Participation of philanthropic foundations or<br>for-profit organizations

C9-2019: "CRISPR/Cas9 and CRISPRi systems for metabolic engineering of bacteria"	151	296	CRISPR: 97.2%<br><br>CRISPR/RNAi combined: 0.7%<br><br>Genome editing platforms combined: 0.7%<br><br>RNAi: 0.7%<br><br>TALENs: 0.7%	Chinese Academy of Science<br><br>National Tsing Hua University (Taiwan)<br><br>University of Illinois Urbana-Champaign	National Science Foundation USA<br><br>Ministry of Science and Technology of Taiwan<br><br>National Institutes of Health USA	Application<br><br>Academic institutions with public funding
C10-2019: "Degradation of dsRNA as a factor of RNAi efficiency in Insect pest control"	101	168	RNAi: 94%<br><br>CRISPR: 5%<br><br>Viral vectors: 1%	Ghent University<br><br>University of Kentucky	Center for Arthropod Management Technologies<br><br>National Institute of Food and Agriculture USA<br><br>National Natural Science Foundation of China	Application<br><br>Academic institutions with public funding
C11-2019: "CRISPR/Cas-based gene drive for insect pest control"	96	179	CRISPR: 91.6%<br><br>RNAi: 4.2%<br><br>TALENs: 2.1%<br><br>Genome editing platforms combined: 1%<br><br>Meganucleases: 1%	Chinese Academy of Science	National Natural Science Foundation of China	Application<br><br>Academic institutions with public funding
C12-2019: "Application of CRISPR/Cas technology in livestock research"	89	112	CRISPR: 82.1%<br><br>RNAi: 9%<br><br>TALENs 5.6%<br><br>Viral vectors 1.1%<br><br>ZFN: 1.1%<br><br>Meganucleases 1.1%	Friedrich Loeffler Institute<br><br>Jilin University<br><br>Northwest A&F University<br><br>ShanghaiTech University	National Natural Science Foundation of China	Application<br><br>Academic institutions with public funding
C13-2019: "Crispr-cas9 based genome editing in fungus species"	85	167	CRISPR: 95.2%<br><br>TALENs 2.4%<br><br>Viral vectors: 1.2%<br><br>RNAi: 1.2%	No leading research institution identified	No leading founding agency identified	Application<br><br>Leading research institutions and funding agencies not identified
C14-2019: "Development of Crispr/Cas9 based HIV-1 therapeutics"			CRISPR: 73.5%<br><br>Viral vectors: 14.5%<br><br>ZFNs: 4.8%<br><br>Genome editing/viral vectors combined: 3.6%<br><br>TALENs: 2.4%<br><br>RNAi: 1.2%<br><br>CRISPR/RNAi combined: 1.2%<br><br>Genome editing platforms combined: 1.2%	Academic Medical Center-University of Amsterdam	National Natural Science Foundation of China<br><br>Chinese Academy of Science	Application<br><br>Academic institutions with public funding
C15-2019: "Deactivated Crispr-cas9 (dCas9)"	76	129	CRISPR: 96.1%<br><br>Viral vectors: 1.3%<br><br>RNAi 2.6%	University of California System<br><br>Harvard University<br><br>Howard Hughes Medical Institute<br><br>Massachusetts Institute of Technology	National Institutes of Health USA<br><br>National Science Foundation USA	Improvement<br><br>Academic institutions with public funding
C16-2019: "Anti-CRISPR phage-encoded proteins as 'off-switch' tools"	70	310	CRISPR: 100%	University of California System<br><br>University of Toronto	National Institutes of Health USA<br><br>Canadian Institutes of Health Research<br><br>National Science Foundation USA<br><br>Sandler Foundation	Improvement<br><br>Participation of philanthropic foundations or for-profit organizations
C17-2019: "Cas9-cytidine deaminase fusion for individual bases editing"	59	111	RISPR: 94.6%<br><br>Genome editing/viral vectors combined: 5.4%	Harvard University<br><br>Broad Institute<br><br>Massachusetts Institute of Technology	National Institutes of Health USA<br><br>Japan Agency for Medical Research and Development<br><br>Japan Society for the Promotion of Science	Improvement<br><br>Academic institutions with public funding
