# Supplementary figures and images for: Mapping the coevolution, leadership and financing of research on viral vectors, RNAi, CRISPR/Cas9 and other genomic editing technologies
Source: PLoS One. 2020 Apr 15;15(4):e0227593. doi: 10.1371/journal.pone.0227593 (PMC7159216; doi:10.1371/journal.pone.0227593)

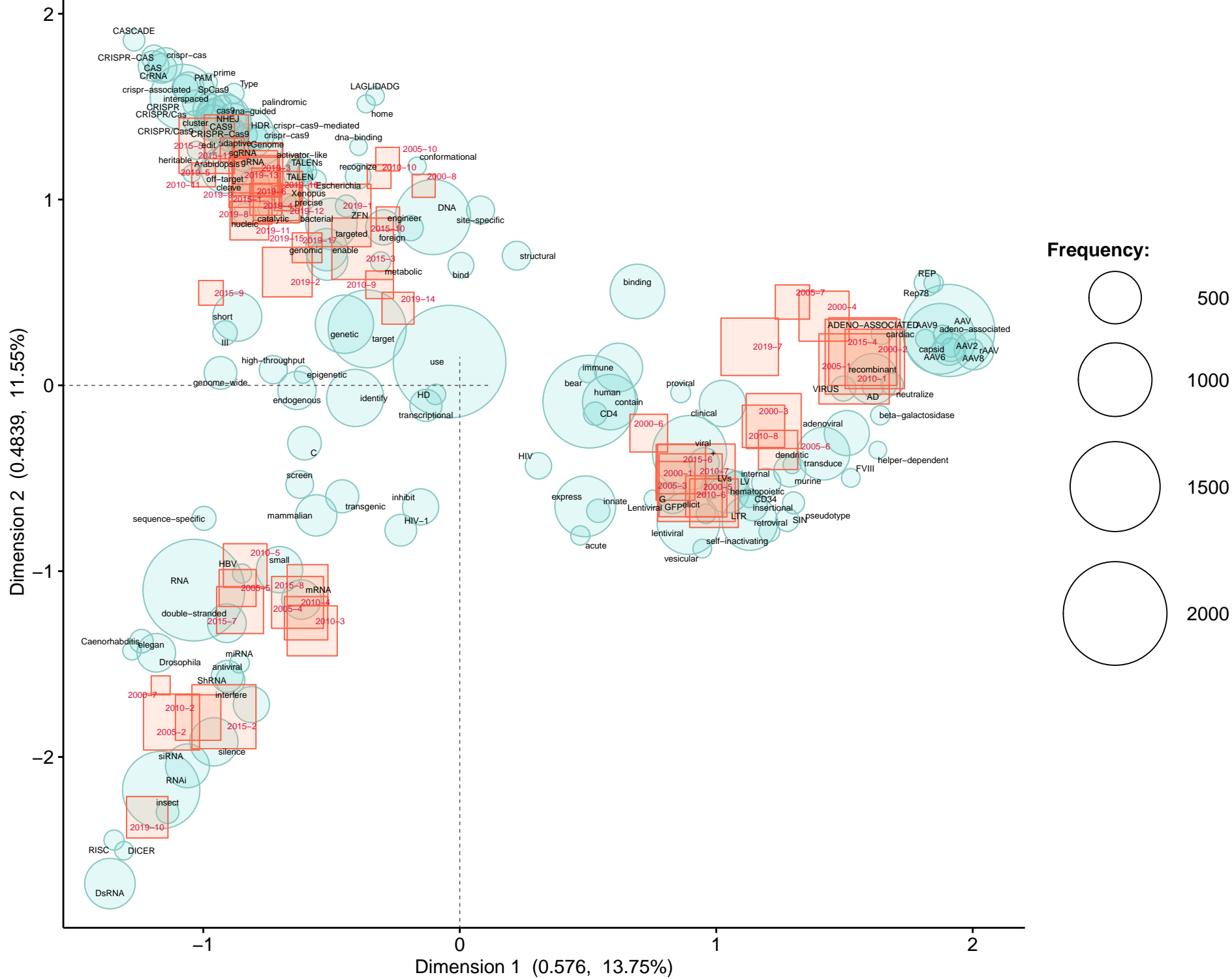

Supplement: S1 Fig — (PDF) [file pone.0227593.s003.pdf]
